# Patterns and environmental drivers of C, N, and P stoichiometry in the leaf‐litter‐soil system associated with Mongolian pine forests

**DOI:** 10.1002/ece3.11172

**Published:** 2024-03-20

**Authors:** Yue Ren, Guang‐lei Gao, Guo‐dong Ding, Ying Zhang, Pei‐shan Zhao

**Affiliations:** ^1^ Yanchi Research Station, School of Soil and Water Conservation Beijing Forestry University Beijing China; ^2^ State Key Laboratory of Efficient Production of Forest Resources Beijing Forestry University Beijing China; ^3^ Engineering Research Center of Forestry Ecological Engineering, Ministry of Education Beijing Forestry University Beijing China; ^4^ Key Laboratory of State Forestry and Grassland Administration on Soil and Water Conservation Beijing Forestry University Beijing China

**Keywords:** ecological stoichiometry, environmental factors, homeostasis; Mongolian pine, nutrient resorption

## Abstract

Ecological stoichiometry is an important approach to understand plant nutrient cycling and balance in the forest ecosystem. However, understanding of stoichiometric patterns through the leaf‐litter‐soil system of Mongolian pine among different stand origins is still scarce. Therefore, to reveal the variations in Mongolian pine carbon (C), nitrogen (N), and phosphorus (P) stoichiometry and stoichiometric homeostasis among different stand origins, we measured C, N, and P concentrations of leaves, litter, and soil, and analyzed the nutrient resorption efficiencies of leaves in differently aged plantations and natural forests from semi‐arid and dry sub‐humid regions. The results showed that (1) the stand origin had a significant effect on the C–N–P stoichiometry, and also significantly affected leaf N and P reabsorption efficiencies. Leaf N/P ratios indicated that Mongolian pine was co‐limited by N and P in the NF, HB and HQ, and was mainly limited by P in MU. (2) With increasing stand age, C concentrations in the leaf‐litter‐soil system initially increased and then decreased, the N and P concentrations and reabsorption efficiencies in the leaf‐litter‐soil system were gradually increased. Overall, stand age had a significant effect on N concentrations, C/N and C/P ratios in the leaf‐litter‐soil system. (3) The C and N elements between the leaf‐litter‐soil system had a strong coupling relationship, and the P element between litter‐soil had a strong coupling relationship. In addition, plantations exhibited greater N/P homeostasis than natural forests, and N/P exhibited greater homeostasis than N and P alone, which may be a nutrient utilization strategy for forests to alleviate N or P limitation. (4) Environmental factors have a significant influence on C–N–P stoichiometry in the leaf‐litter‐soil system, the most important soil properties and meteorological factors being soil water content and precipitation, respectively. These results will be essential to provide guidance for plantation restoration and management in desert regions.

## Graphical abstract

Stand origin significantly affects the C–N–P stoichiometry in the leaf‐litter‐soil system and leaf N and P reabsorption efficiency of the Mongolian pine. The N/P homeostasis of the plantation is greater than that of the natural forest. Environmental factors have a significant impact on the C–N–P stoichiometry of the Mongolian pine, particularly soil water content and precipitation.

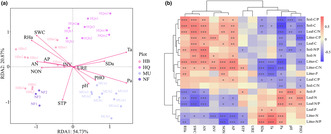

## 1 INTRODUCTION

Ecological stoichiometry is a comprehensive approach to cognitive energy balance and element limiting in biogeochemical processes (Elser et al., 2010; Yu et al., 2020). Carbon, nitrogen, and phosphorus are indispensable elements for forest succession, production and sustainable management in the terrestrial ecosystem (Chen et al., 2004; Li et al., 2017). Carbon (C) is the basic structural and energy element, constituting one‐half dry biomass of plant (Sardans & Peñuelas, 2012). Nitrogen (N) and phosphorus (P) are the major components of various proteins and genetic materials involved in the process of cell synthesis and division, photosynthesis and N‐fixation, as they are essential mineral elements for the growth of plants (Michaels, 2003; Wang et al., 2023; Zhang et al., 2021). Ecological stoichiometry reveals the functioning of the terrestrial ecosystem and the N or P environmental adaptation strategies of plants by discussing elemental ratios (Ågren, 2004; Elser & Urabe, 1999; Gao et al., 2019).

As the most physiologically active organs in forest ecosystems, leaves respond to plant nutrient requirements, soil nutrient supply and environmental changes (Wang & Zheng, 2020). Litter is a key biota link between aboveground and belowground, and its decomposition process is an important way to maintain the material circulation and nutrient balance in the forest ecosystem (Zhang, Liu, et al., 2019; Zhang, Zhang, et al., 2019). More than half of the net production of the above‐ground portion returns to the ground as litter, which then returns nutrients to the soil for uptake and utilization by plants through the decomposition by decomposers and soil microorganisms (McGroddy et al., 2004; McNaughton et al., 1989). Soil is an important component and nutrient pool of forest ecosystems, plant nutrients mainly come from soil, and part of the nutrients can be replenished through plant litter (Cusell et al., 2014; Dong et al., 2019). Therefore, the stoichiometry characteristics of elements in leaf‐litter‐soil are crucial to the modeling of plant nutrient availability and elemental biogeochemical cycling in terrestrial ecosystems (Ge & Xie, 2017).

Concentrations of nutrient elements in leaves, litter and soil show obvious pattern of temporal and spatial variation (Bui & Henderson, 2013). A series of abiotic (e.g., elevation, climate, and soil property) and biotic factors (e.g., stand age) have been shown to influence the C, N, and P stoichiometry in leaf‐litter‐soil system over broad geographical scales (Ge & Xie, 2017). The stoichiometric ratios of elements in the environment affect those of organisms in their environment, while organisms also affect the elemental composition of their environment by absorbing or releasing elements in ratios that are different from ambient ratios (Normand et al., 2017). The material circulation and energy fluxes in terrestrial ecosystems are indirectly influenced by soil physicochemical properties. The relationship between plant C, N, and P stoichiometry and the environment may reflect the adaptability of the plant to the environmental changes (Yang et al., 2019). The forest microenvironment changes continuously with increasing age. Consequently, the spatiotemporal pattern of C–N–P stoichiometric characteristics in forest ecosystems is facilitated by the dynamic changes of C, N, and P with respect to stand age and space.

Forests play a vital role in maintaining ecological environmental balance, biodiversity, and soil and water conservation (Song et al., 2011). As a major component of the world's forest resources, plantations play a role in restoring and rebuilding the ecological environment (Ren et al., 2023; Sun et al., 2017), especially in the northern desert areas that are heavily impacted by wind erosion (Zhao et al., 2020). Mongolian pine is a strongly cold‐resistant and drought‐resistant tree species that can grow healthily in infertile soils, although its natural distribution range is narrow (Ren et al., 2022; Song et al., 2015). It has become one of the optimal conifer species for constructing protective afforestation in the desert areas of northern China. Herein, to explore the C, N, and P stoichiometry characteristics in leaves, litter and soil of Mongolian pine, three different‐age plantations (24–43 a) from different introduction sites and a natural forest were identified as objects. Specifically, the main objective was to figure out the nutrient strategy and limitation of the Mongolian pine from different origins. We hypothesized that (1) C–N–P stoichiometric characteristics in leaf‐litter‐soil may be varied with stand origin and stand age, (2) Mongolian pine has a stoichiometric homeostasis that differs in forests, and (3) the stand origin and stand age impacted the C, N, and P stoichiometric characteristics through the variations in climate and soil environment.

## 2 MATERIALS AND METHODS

### 2.1 Experimental design and study sites

**FIGURE 1:**
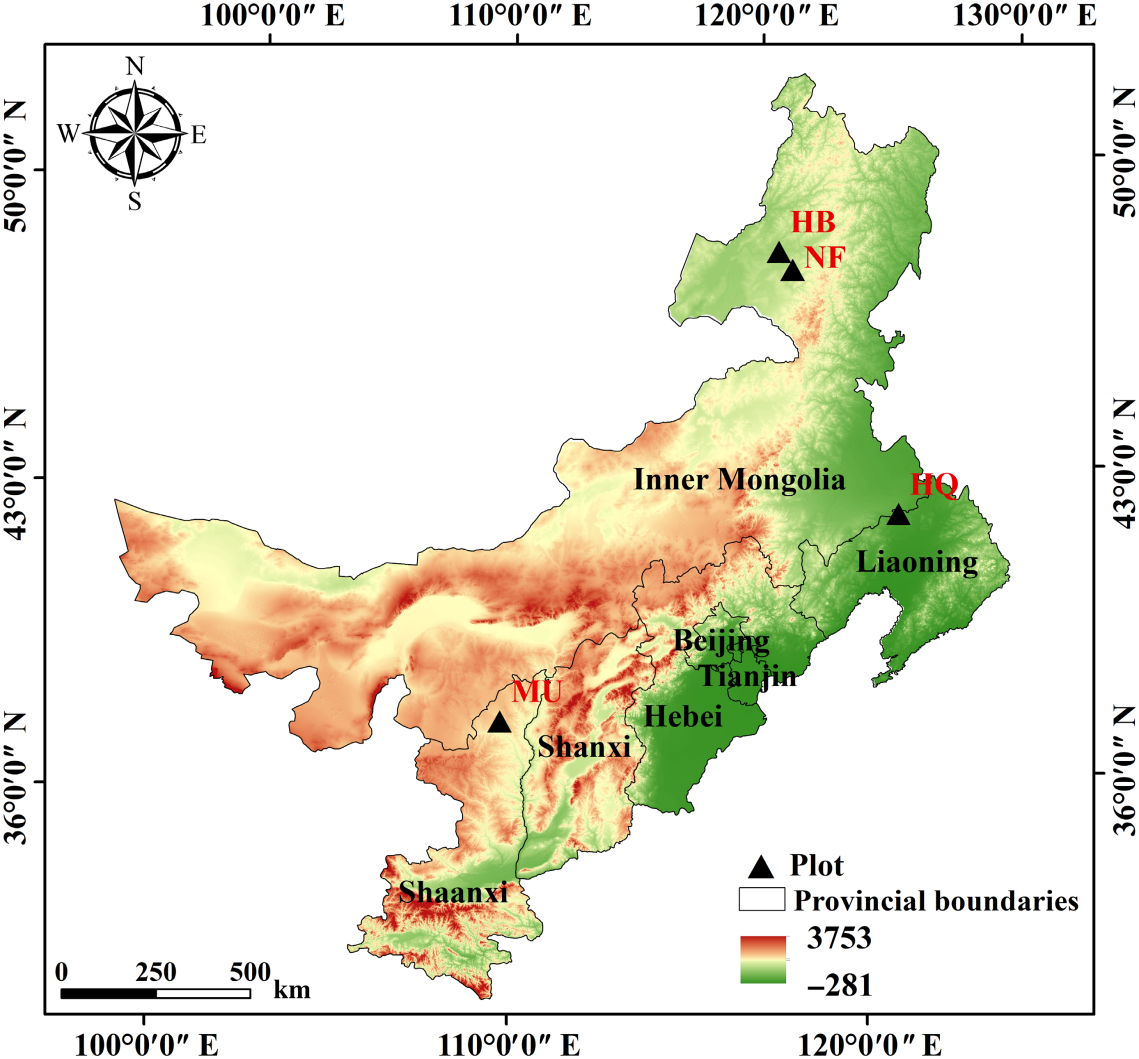
Sampling locations of study sties. HB, the plantations in Hulunbuir Desert; HQ, the plantations in Horqin Desert; MU, the plantations in Mu Us Desert; NF, natural forest. The same below.

The study sites are located in the origin and introduction areas of Mongolian pine in northeast China. The natural forest is located in the Honghuaerji National Forest Park (47°36′ N, 115°58′ E; 606 m). The introduction sites were located in Hailar Forest Park (49°07′ N, 119°21′ E; 610 m), Zhanggutai Sandy Land National Forest Park (42°37′ N, 122°22′ E; 226 m) and Hongshixia Sandy Botanical Park (38°16′ N, 109°12′ E; 1080 m). We selected three plantations that used the same silvicultural method and were undisturbed by human activity. The soil (depth at 0–20 cm) in the study areas are both classified as eolian soil, which is highly erodible. The climatic characteristics of each site are shown in Table 1 (1996–2016a). Honghuaerji National Forest Park and Hailar Forest Park are located in the southeast of the Hulunbuir Desert, which has a semi‐arid continental monsoon climate. Hulunbuir Desert is the origin area of Mongolian pine, with an area of 6400 km^2^. The dominant afforestation species are Mongolian pine and *Betula platyphylla*. The dominant shrubs are *Salix cheilophila, Artemisia desertorum, Lespedeza bicolor*, *Leymus chinensis* and *Stipa grandis*. The Zhanggutai Sandy National Land Park is located in the southeast of the Horqin Desert, which has a dry sub‐humid continental monsoon climate. Horqin Desert is the largest sandy land in China, with an area of 63,600 km^2^, which is the first introduction area of Mongolian pine in China. The dominant afforestation species are Mongolian pine (*P*. *sylvestris*), *P. tabuliformis*, and *Populus simonii*. The dominant shrubs are *Cares duriuscula*, *Salsola collina* and *Potentilla anserine*. Hongshixia Sandy Botanical Park is located in the southeast of the Mu Us Desert, which is a semi‐arid continental monsoon climate. Mu Us Desert is one of the most important introduction areas of Mongolian pine in China, with an area of 42,200 km^2^. The dominant afforestation species are Mongolian pine, *Caragana korshinskii* and *P. tabuliformis*. The dominant shrubs are *Setaria viridis*, *Hedysarum scoparium*, and *Amorpha fruticosa* (Figure 1).

**TABLE 1:** Basic information of the plots.

Sandy land	Climate	Plot		Age/a	Average height/m	Average BH/cm	Canopy density	Mean annual temperature/°C	Mean annual precipitation/mm	Mean annual sunshine times/h
Hulunbuir Desert	Semi‐arid continental monsoon climate	NF		6–56	—	—	0.83	0.86	301.51	228.53
		HB	HBh	24	11.98 ± 1.62	14.66 ± 3.40	0.75	−0.78	348.40	227.19
			HBn	33	12.63 ± 1.71	18.36 ± 2.42	0.82			
			HBm	42	13.40 ± 2.36	22.74 ± 1.98	0.71			
Horqin Desert	Dry sub‐humid continental monsoon climate	HQ	HQh	26	10.26 ± 1.47	16.93 ± 2.81	0.72	8.19	511.31	210.92
			HQn	33	10.61 ± 1.03	14.06 ± 2.44	0.75			
			HQm	43	11.12 ± 1.74	21.07 ± 1.02	0.68			
Mu Us Desert	Semi‐arid continental monsoon climate	MU	MUh	26	12.48 ± 3.69	11.76 ± 3.72	0.79	9.47	501.21	232.34
			MUn	32	13.95 ± 2.38	13.58 ± 3.44	0.86			
			MUm	43	14.14 ± 1.84	14.92 ± 3.52	0.73			

Abbreviations: h, half‐mature forest; HB, the plantations in Hulunbuir Desert; HQ, the plantations in Horqin Desert; m, mature forest; MU, the plantations in Mu Us Desert; n, near‐mature forest; NF, natural forest. The same below.

### 2.2 Sampling collection and measurements

Leaf, litter, and soil samples were collected during the peak growth period of plants in July–August, 2017. Three plots were selected from Mongolian pine plantations with different age groups (half‐mature, near‐mature and mature), and the natural forest of Mongolian pine was used as a control (Table 1). The plantation plots had similar site conditions and no manual management. Three replicate plots (50 m × 50 m) were randomly established in each forest. Within each plot, three similar healthy individuals were randomly selected to represent heterogeneity. Then, in order to reduce sampling error, the samples of three standard trees were thoroughly mixed into one sample. To standardize the sample collection, the mature leaves were collected in four directions from individuals. Litter biomass was collected in four directions from the ground. After removal of surface litter and roots, soil samples were taken from a depth of 0–20 cm by using a soil auger. All fresh leaf and litter samples were dried at 105°C for 15 min and then oven‐dried at 70°C to reach constant weight. All soil samples were passed through a 0.25‐mm mesh to remove gravel and plant debris, and then divided into two parts. A portion of soil samples was air dried and passed through a 0.15‐mm mesh for determination of organic carbon, total nitrogen and phosphorus concentrations, while the other fresh soil was stored at 4°C until determined soil enzyme activity and soil available nutrients. Organic carbon concentrations in leaf, litter, and soil samples were determined by potassium dichromate volumetric method (Bao, 2010). To determine the total N and P concentrations, leaf and litter samples were initially digested with H_2_SO_4_–H_2_O_2_, while dry soil samples were digested with H_2_SO_4_–HClO_4_; and then total N and P concentrations followed the kjeldahl acid‐digestion method and molybdenum‐blue method by using an auto element analyzer (Smartchen 450, AMS, Guidonia, Italy). C, N, and P concentrations are expressed as mass concentrations (g/kg), and C/N, C/P, and N/P ratios are expressed as mass ratios.

### 2.3 Climate and soil characteristics

Meteorological data were obtained from the China Meteorological Information Date Service Center (CMDC, http://cdc.cma.gov.cn, Table S2). The data we used included annual sunshine duration (SDa), annual temperature (Ta), annual precipitation (Pa), and annual relative humidity (RHa). Soil pH was measured at a soil: water ratio of 1:2.5 using a PHS‐3E pH meter (INESA, Shanghai, China). Soil water content (SWC) and total porosity (STP) were measured by the weight method. Soil available N (AN), ammonium nitrogen (NON), and available P (AP) were measured using an automatic elemental analyzer (Smartchen 450, AMS, Guidonia, Italy). Soil enzyme activities of invertase (INV), urease (URE), and phosphatase (PHO) were determined using the method according to Cao et al. (2021).

### 2.4 Statistical analysis

Leaf nutrient resorption efficiency (NuRE) was calculated as follows:

$$\text{NuRE}=\frac{N_{g}-N_{l}\times \text{MLCF}}{N_{g}}\times 100\%,$$

where *N*_g_ is the nutrient concentration of N and P in fresh leaves, *N*_l_ is the nutrient concentration of N and P in litter (Wang et al., 2021), and MLCF is mass loss correction factor. Corrections for nutrient reabsorption rates are needed since external modifications such as area wrinkles occur during leaf senescence, and the mean value of 0.832 for the major woody plants in northern China was used as the correction factor.

The degree of stoichiometric homeostasis of Mongolian pine was characterized by homeostasis coefficient *H* (Persson et al., 2010). Since the slope of the regression (1/*H*) was generally considered to be greater than or equal to 0, one‐tailed tests with *α* < 0.1 were used. If the regression was no significant (*α* > 0.1), the organism was considered to be ‘strictly homeostatic’. All datasets with significant regressions were classified as 0 < 1/*H* < 0.25 ‘homeostatic’, 0.25 < 1/*H* < 0.5 ‘weakly homeostatic’, 0.5 < 1/*H* < 0.75 ‘weakly plastic’, or 1/*H* > 0.75 ‘plastic’ (Bai et al., 2019). For 1/*H* > 1, 1/*H* close to 1 indicates weak or no stoichiometric homeostasis, and 1/*H* much larger than 1 indicates ‘homeostatic’.

One‐way analysis of variance (ANOVA) and least significant difference (LSD) multiple comparisons were used to determine the differences in the C–N–P stoichiometry and nutrient resorption efficiency among different stand origins and stand ages. Multivariate ANOVA with post‐hoc Tukey's tests was used to determine the effect of the stand origin, stand age, and their interaction on the C–N–P stoichiometry and nutrient resorption efficiency. Person's correlation was performed to show the relationship between the C–N–P stoichiometry in the leaf‐litter‐soil system. The correlation heatmap reveals the relationship between environmental factors and the C–N–P stoichiometry. Redundancy analysis (RDA) was performed to determine the environmental variables affecting the C–N–P stoichiometry. Nonmetric multidimensional scaling ordination (NMDS) with permutational multivariate analysis of variance (PERMANOVA) was performed to determine the contribution of stand origin and stand age to the C–N–P stoichiometry. Values are expressed as the mean ± standard deviation (SD). All statistical analyses were performed with SPSS 21.0 (SPSS Inc., Chicago, IL, USA) and R software (Version 4.3.0) at a significance level of 0.05.

## 3 RESULTS

### 3.1 C–N–P stoichiometry and nutrient resorption in leaf

**FIGURE 2:**
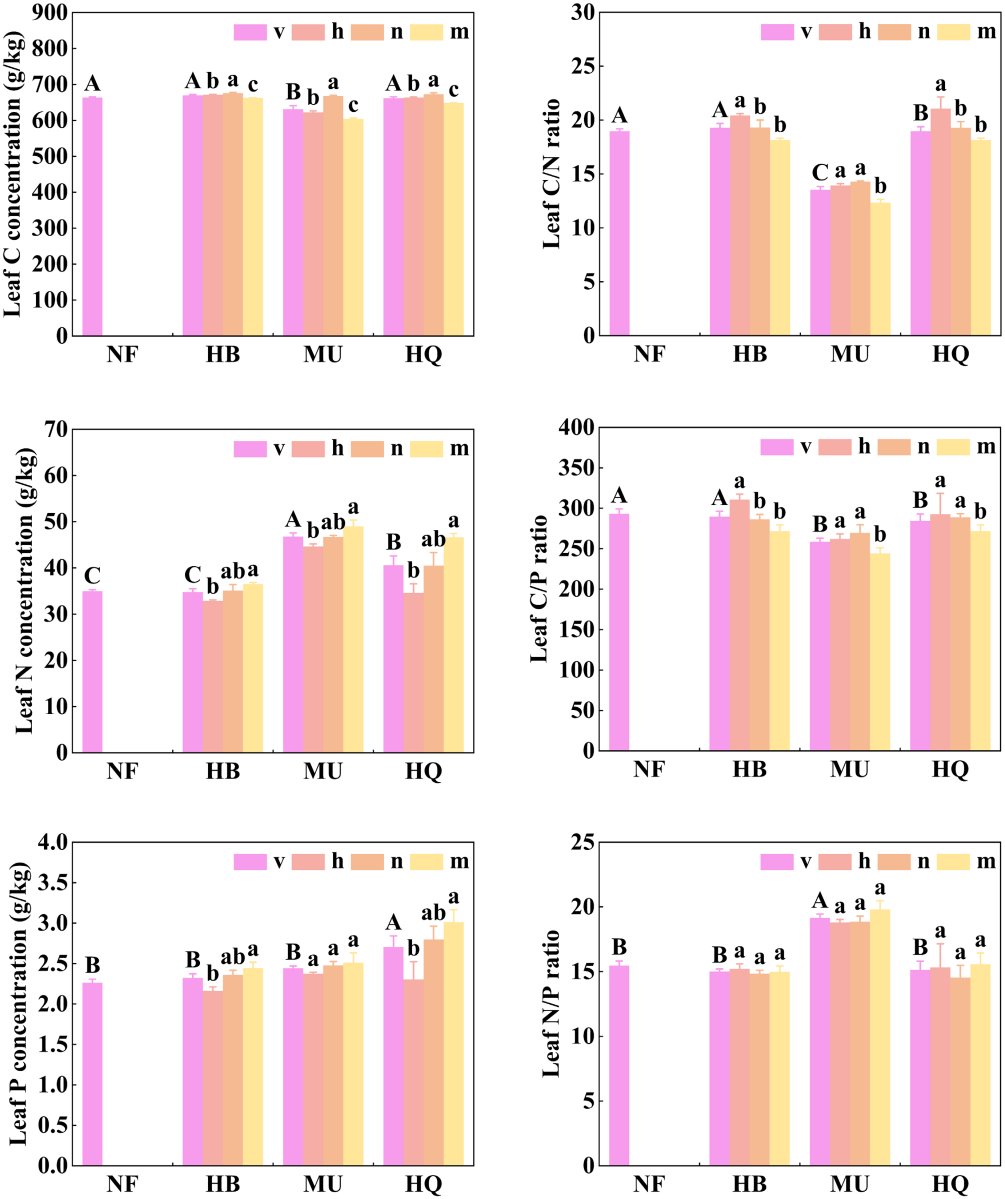
Differences in leaf C, N, and P stoichiometric characteristics across the growth stage between natural forest and plantations. Error bars are the standard error (*n* = 3). “h” for the half‐mature forest, “n” for the near‐mature forest, “m” for the mature forest, “v” for the mean value of h, n, and m. Different capital letters indicate significant differences in mean values among study areas, different litter letters indicate significant differences among stand ages.

Stand origin had a significant effect on leaf C concentrations (*p* < .05, Table 2), although stand age had no significant effect (*p* > .05, Table 2), with an initial increasing and then decreasing trend (Figure 2). Stand origin and stand age have a significant effect on leaf N, P concentrations and C/N, C/P ratios (*p* < .05, Table 2). Leaf N and P concentrations were significantly lower in NF and HB than in HQ. Leaf N and P concentrations tended to increase in the process of stand maturation (Figure 2). Leaf C/N and C/P ratios in NF and HB were significantly higher than those in HQ and MU (Figure 2). Age pattern of C/N/P varied across stand origins. There was no significant difference in leaf N/P among the stand ages (Table 2). Leaf C/N and C/P ratios showed a downward trend with stand maturation in HB and HQ (Figure 2). Leaf N/P ratios ranged from 14 to 16 in NF, HB, and HQ, and were more than 16 in MU.

**TABLE 2:** Two factors variance analysis of effect of stand origin and stand age on the C–N–P stoichiometry and nutrient resorption efficiency.

Component	Variable	Stand origin		Stand age		Stand origin*stand age		Stand age (stand origin)	
		*F*	*p*	*F*	*p*	*F*	*p*	*F*	*p*
Leaf	C	6.975	**.006**	2.347	.124	6.573	**.002**	80.361	**.000**
	N	57.972	**.000**	18.027	**.000**	2.914	.051	7.952	**.000**
	P	8.868	**.002**	8.241	**.003**	1.936	.148	4.038	**.010**
	C:N	70.644	**.000**	20.665	**.000**	3.205	**.038**	9.025	**.000**
	C:P	9.964	**.001**	11.500	**.001**	2.266	.102	5.344	**.000**
	N:P	24.612	**.000**	0.548	.587	0.202	.934	0.317	.920
	NRE	95.135	**.000**	4.232	**.031**	1.221	.337	2.224	.088
	PRE	30.228	**.000**	10.124	**.001**	16.461	**.000**	13.349	**.000**
Litter	C	348.598	**.000**	53.488	**.000**	1.751	.183	18.996	**.000**
	N	153.909	**.000**	15.052	**.000**	4.069	**.016**	9.662	**.001**
	P	6.975	**.006**	2.347	.124	6.975	**.000**	5.164	**.003**
	C:N	250.726	**.000**	17.330	**.000**	0.897	.486	6.374	**.001**
	C:P	27.921	**.000**	15.263	**.000**	7.747	**.001**	10.268	**.000**
	N:P	60.756	**.000**	0.042	.959	1.661	.959	1.121	.389
Soil	C	250.571	**.000**	90.986	**.000**	41.844	**.000**	58.225	**.000**
	N	65.423	**.000**	29.175	**.000**	5.591	**.004**	13.452	**.000**
	P	4.396	**.028**	0.472	.631	1.045	.412	0.854	.546
	C:N	111.111	**.000**	104.702	**.000**	26.478	**.000**	52.553	**.000**
	C:P	229.276	**.000**	81.049	**.000**	81.049	**.000**	51.395	**.000**
	N:P	297.030	**.000**	0.981	.394	9.964	**.000**	6.970	**.001**

*Note*: The bold values indicates a significant effect.

Abbreviations: NRE, N resorption efficiency; PRE, P resorption efficiency.

**FIGURE 3:**
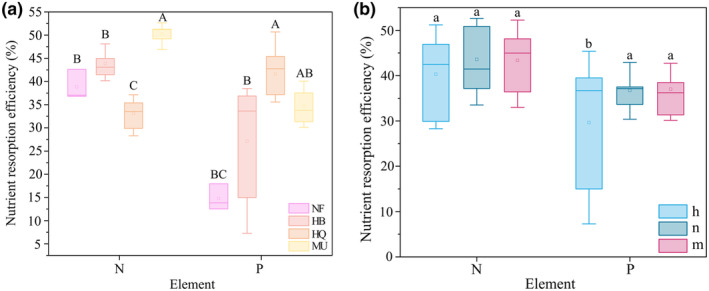
N and P resorption efficiencies of Mongolian pine in different stand origin (a) and stand age (b). Different capital letters indicate significant differences among study areas, different litter letters indicate significant differences among stand ages.

Stand origin had a significant effect on N and P resorption efficiencies (*p* < .05, Table 2). N resorption efficiencies were significantly higher in MU than in NF and other plantations. P resorption efficiencies were significantly lowest in NF (Figure 3a). N resorption efficiency was significantly higher than that of P in all but except for HQ. Stand age significantly affected both N and P resorption efficiencies (*p* < .05), with an increasing trend (Figure 3b).

### 3.2 C–N–P stoichiometry in litter

**FIGURE 4:**
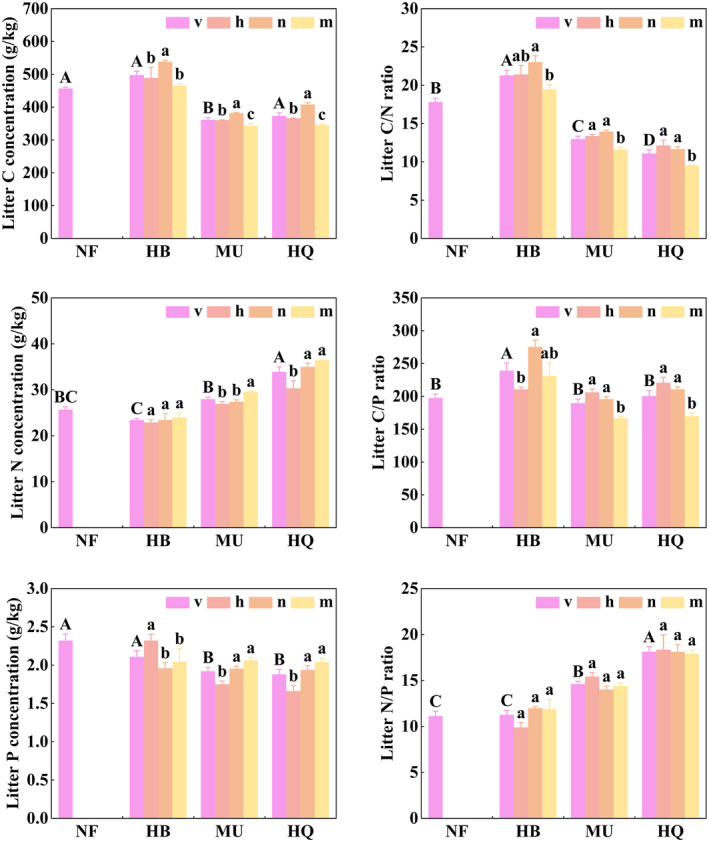
Differences in litter C, N, and P stoichiometric characteristics across the growth stage between natural forest and plantations. Error bars are the standard error (*n* = 3). “h” for the half‐mature forest, “n” for the near‐mature forest, “m” for the mature forest, “v” for the mean value of h, n, and m. Different capital letters indicate significant differences in mean values among study areas, different litter letters indicate significant differences among stand ages.

Stand origin and stand age had a significant effect on litter C, N concentrations, and C/N, C/P ratios (*p* < .05, Table 2). Litter C concentrations were significantly higher in NF than in MU, but N concentrations were significant lower than in HQ (Figure 4). Litter C/N and C/P ratios were markedly higher in NF than in HB (Figure 4). In plantations, litter C concentrations were highest in near‐mature forests, whereas litter C/N and C/P ratios were relatively low in mature forests. Stand origin had a significant effect on litter P concentrations and N/P ratios, whereas stand age had no significant effect (*p* > .05, Table 2). In HQ and MU, litter P concentrations tended to increase with stand maturation (*p* < .05, Figure 4).

### 3.3 C–N–P stoichiometry in soil

**FIGURE 5:**
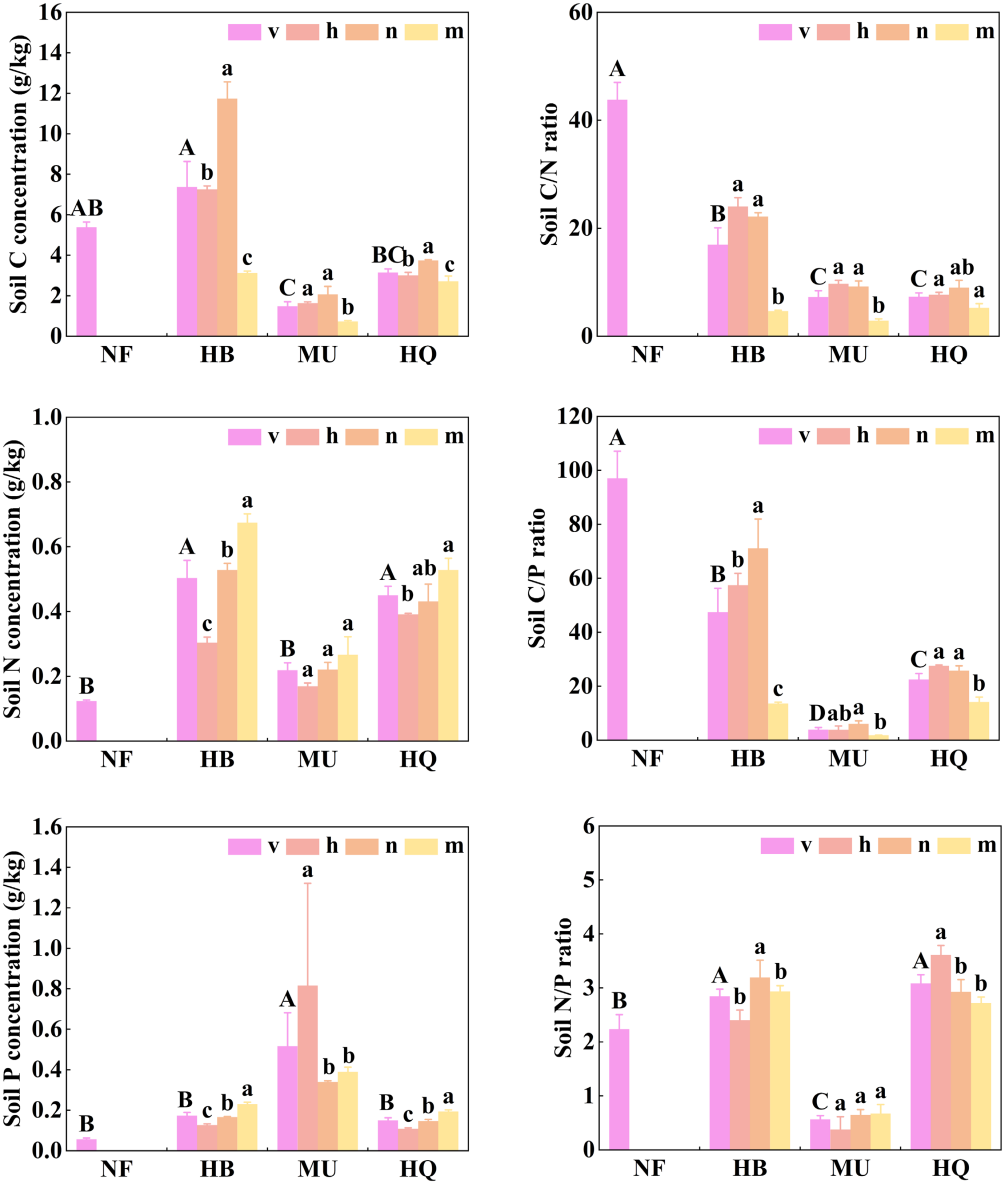
Differences in soil C, N and P stoichiometric characteristics across the growth stage between natural forest and plantations. Error bars are the standard error (*n* = 3). “h” is for the half‐mature forest, “n” for the near‐mature forest, “m” for the mature forest, “v” for the mean value of h, n, and m. Different capital letters indicate significant differences in mean values among study areas, different litter letters indicate significant differences among stand ages.

Soil C–N–P concentrations were significantly affected by stand origin (*p* < .05, Table 2). Soil C concentrations were higher in NF than in HQ and MU. In contrast to C, soil P concentrations were higher in natural forests than in introduced forests. Stand age had minimal effects on soil P concentrations, but significant effects on soil C and N concentrations (*p* < .05, Table 2). In plantations, soil N concentrations accumulated with stand maturation (Figure 5). Differences in soil P concentrations among the plantations were not significant, ranging from 0.13 to 0.39 g/kg. Stand origin and stand age had a significant effect on soil C/N and C/P ratios (*p* < .05, Table 2). Soil C/N and C/P ratios were markedly higher in natural forests than in introduced forests, and for plantations were lowest in mature forests (Figure 5). Overall, the differences in soil C/N/P were obvious between the natural forest and introduced plantations.

### 3.4 Linkages among C–N–P stoichiometry in leaf‐litter‐soil and stoichiometric homeostasis

**FIGURE 6:**
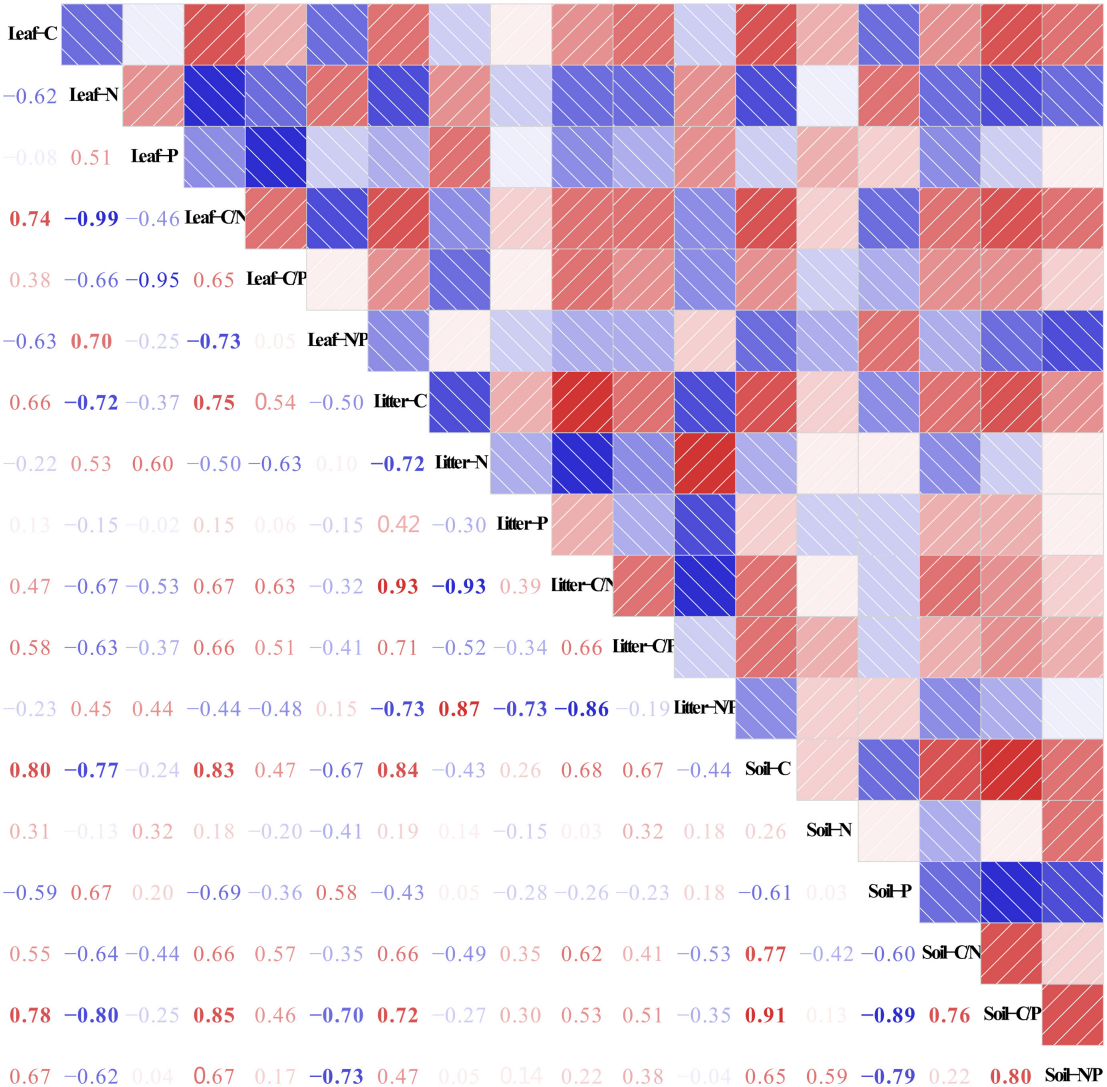
Relationship between C–N–P stoichiometric characteristics among leaf‐litter‐soil system. Red indicates positive correlations, blue indicates negative correlations. The numbers in the graph are correlation coefficients.

The C–N–P stoichiometric characteristics exhibited a slightly different relationship among the leaf‐litter‐soil system (Figure 6). Remarkable significant positive relationships were detected in C concentration, C/N and C/P ratios among leaf‐litter‐soil (*p* < .01, Table S1). In the leaf‐litter‐soil system, C concentrations were significantly negatively correlated with N concentration (*p* < .05), but significantly positively correlated with C/N ratios (*p* < .05). N concentration significantly positively correlated with N/P ratio (*p* < .05), but significantly negatively correlated with C/N and C/P ratios (*p* < .05). Furthermore, N concentrations were significantly negatively correlated with P concentration in leaf and soil (*p* < .05), but not in litter.

Leaf stoichiometric homeostasis varied by forest origin. Differences exist between natural forest and plantations, as well as between N, P and N/P (Table 3). We found strict homeostasis of N and P concentrations in NF and MU (*p* > .1) and homeostatic in HB. In HQ, however, N concentrations were weakly plastic and P concentrations were weakly homeostatic. N/P were homeostatic in NF, whereas N/P were strictly homeostatic in plantations.

**TABLE 3:** Regression slopes (1/*H*) of relationships between plant and soil nutrient stoichiometry in natural forest and plantations of the Mongolian pine.

	Variable		*I*/*H*	*R* ^2^	*p*	Degree
	*x*	*y*				
NF	Soil‐N	Leaf‐N	−0.225	.431	.544	Strictly homeostatic
	Soil‐P	Leaf‐P	−0.005	.001	.928	Strictly homeostatic
	Soil‐N/P	Leaf‐N/P	−0.103	.328	.623	Homeostatic
HB	Soil‐N	Leaf‐N	0.115	.528	.079	Homeostatic
	Soil‐P	Leaf‐P	0.196	.624	.047	Homeostatic
	Soil‐N/P	Leaf‐N/P	−0.085	.094	.423	Strictly homeostatic
HQ	Soil‐N	Leaf‐N	0.718	.753	.003	Weakly plastic
	Soil‐P	Leaf‐P	0.475	.597	.015	Weakly homeostatic
	Soil‐N/P	Leaf‐N/P	0.327	.146	.311	Strictly homeostatic
MU	Soil‐N	Leaf‐N	0.077	.185	.420	Strictly homeostatic
	Soil‐P	Leaf‐P	−0.009	.021	.478	Strictly homeostatic
	Soil‐N/P	Leaf‐N/P	0.145	.043	.592	Strictly homeostatic

### 3.5 Factors affecting C–N–P stoichiometry in leaf‐litter‐soil system among different stand origin and stand age

**FIGURE 7:**
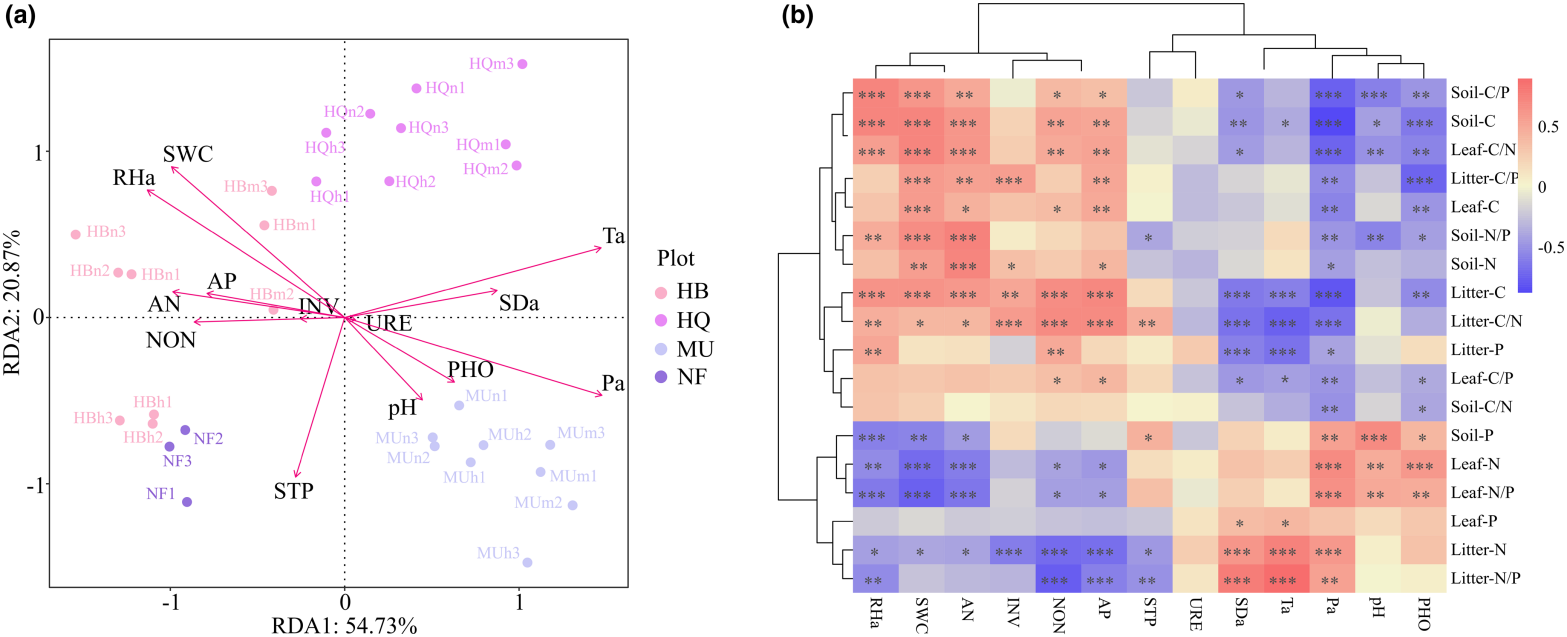
Relationship between C–N–P stoichiometric characteristics and environmental factors, including RDA analysis (a) and correlation analysis (b). The arrow direction and length indicate correlation to C–N–P stoichiometry and effect size of the variables, and asterisks and double asterisks indicate *p* < .05 and *p* < .01, respectively.

Stand origin and stand age impacted the C–N–P stoichiometric characteristics through the variations in climate and soil environment (Figure 7, Figure S1). Redundancy analysis (RDA) revealed that the meteorological and soil properties explained 75.6% of the total variation in the date, with axes 1 and 2 explaining 54.73% and 20.87% of the total variation, respectively (PERMANOVA, *F* = 7.329, *p* = .001, Figure 7a). Expect INV and URE, climate and soil physicochemical properties had notably effects on the C–N–P stoichiometry (*p* < .01, Table S2), among them, the factors that contributed most were Pa and SWC, respectively. The environmental factors mentioned in this study play different roles in different sample sites. RHa and SWC were overall positively related to C–N–P stoichiometry in HB, and were negatively related to C–N–P stoichiometry in MU (Figure 7a). Ta was overall positively related to C–N–P stoichiometry in HQ, and were negatively related to C–N–P stoichiometry in NF (Figure 7a).

The correlation heatmap showed that soil available nutrients (AP, AN, NON) were positively related to leaf N, leaf C/N and litter N, but were negatively related to leaf C, leaf C/N, litter N and litter C/N (*p* < .05, Figure 7b). SWC was positively related to litter C/N and C/P. SDa and Ta were positively related to litter N and N/P. Pa and PHO were negatively related to soil C/N, C/P and N/P (*p* < .01, Figure 7b). URE and plant‐litter‐soil C‐N‐P stoichiometry were not significantly correlated (*p* > .05, Figure 7b).

## 4 DISCUSSION

### 4.1 C–N–P stoichiometry in leaf‐litter‐soil system among the stand origin and stand age

Plant ecological stoichiometry is closely related to the environment (Feng et al., 2017), resulting in significant differences in leaf nutrient concentrations and C/N/P ratios, which was consistent with our first hypothesis. In this study, leaf C, N, and P concentrations were higher than the global averages (464, 20.9 and 1.99 g/kg) (Elser et al., 2000), suggesting that the Mongolian pine ecosystem is enriched in C, N, and P, proving that plants from resource‐deficient environments have a greater nutrient storage capacity than plants from resource‐sufficient environments (Chapin et al., 1990). The data presented herein showed that the stand age had no significant effect on leaf C concentration. At first sight, this may seem strange, but it makes sense, because plant C is not directly involved in production activities, instead, it mainly acts as a relatively stable structural basis of plants (Zhang, Gao, et al., 2018; Zhang, Wang, et al., 2018). Leaf C concentration first increased and then decreased with increasing stand age, which was caused by the difference in plant growth rate. Plants grew rapidly at mid‐ and near‐maturity, with constant dry matter synthesis and storage, but growth gradually slowed down during maturity. Plant C/N and C/P reflect the nutrient use efficiency of plant growth rate (Wang & Zheng, 2020; Yu et al., 2017). Our results showed that leaf C/N and C/P ratios were lowest in mature forest, possibly because the N, P concentrations and competitiveness increased with stand maturity. Leaf N/P ratios were highest in MU, which was consistent with a meta‐analysis of leaf N/P mass ratios on a global scale, i.e. leaf N/ P ratios increased with increasing temperature (Reich & Oleksyn, 2004). In addition, leaf N/P ratios were proposed to assess N or P limitation (Cleveland & Liptzin, 2007; Mayor et al., 2017). The N/P threshold theory has shown that N/P < 14 is the limit of N and N/P > 16 is the limit of P. Between 14 and 16 indicates co‐limitation of N and P (Koerselman & Meuleman, 1996). Leaf N/P ratios in NF, HB and HQ were in the range of 14 to 16, indicating N and P co‐limitation for Mongolian pine growth, but leaf N/P in MU were higher than 16, indicating P limitation over time. These results not only support the hypothesis that P becomes increasingly limiting relative to N in forests (Deng et al., 2019; Hayes et al., 2014), but also confirm that natural forests at high latitudes are generally limited by P (Elser et al., 2007).

Litter plays a critical role in the nutrient cycling of terrestrial ecosystems (Sterner & Elser, 2002), which can not only enhance the structural heterogeneity of forest ecosystems but also increase ecosystem biodiversity (Sulkava & Huhta, 1998). Compared to global plants, desert plants had higher litter N and P concentrations (global level, 10.9 and 0.85 g/kg) (Kang et al., 2010), consequently, litter C/N, C/P and N/P ratios were lower than the global average (50.1, 659 and 420.3) (McGroddy et al., 2004). In accordance with our first hypothesis, stand origin and stand age significantly affected the variation in litter C/N and C/P ratios, confirming that litter decomposition is susceptible to the influence of internal plant changes and the external environment. Interestingly, litter N/P showed little difference with stand age, indicating that the degree of decomposition, decomposition rate and nutrient return rate of litter with a relatively balanced during plant growth. Litter C/N can reflect the organic matter decomposition and N release (Xu et al., 2022), and it is generally accepted that higher C/N implies slower decomposition rate and nutrient release (Enríquez et al., 1993; Liu & Wang, 2021). Our study has reported that the lowest litter C/N ratio occurred in HQ, indicating that the litter return accelerated litter decomposition and nutrient turnover in the Mongolian pine plantation. Moreover, the litter C/N and C/P ratios were lowest in mature forests, demonstrating that the litter is highly humified and easily decomposed, and thus can rapidly supplement soil nutrients.

As a medium for plant growth, soil is the largest reservoir of nutrients for plant growth. In our study, the soil C, N and P concentrations were much lower than the Chinese average level, suggesting that sandy soils are poor in nutrient retention. It is related to the growth habit and litter decomposition of Mongolian pine. Firstly, conifers often accumulate in N‐deficient soils, and secondly, the acidic environment formed by the decomposition of conifer litter largely inhibits the activities of microorganisms, which is not conducive to soil nutrient accumulation (Aerts & Chapin, 2000). In addition, soil C/N ratios in this study were lower than average level in China (10.0–12.0) and global (13.33) (Cleveland & Liptzin, 2007; McGroddy et al., 2004; Tian et al., 2010). Similarly, soil C/P and N/P ratios were lower than average level in China (61.0 and 5.2) (Zhu et al., 2013). As predicted, soil C–N–P stoichiometric characteristics showed spatial variability. Soil P concentration was highest in the MU, because the soil P concentration shows an increasing trend from semi‐humid area to semi‐ arid and arid area (Zhou & Zhu, 2003). It is worth mentioning that soil P concentrations were relatively stable across stand ages, which was contrary to our predictions. This perhaps due to the different sources of soil P. Compared with C and N, P is the parent material of the soil and phosphate decomposition takes a long period of time (Deng et al., 2019). Our data show that soil N and P concentrations increased with the stand aging. The main reason is that the forest community tends to become more complex and diverse during stand development, and the decomposition of soil animals and microorganisms enhances the input of organic compounds. Soil C/N/P ratios are a diagnostic indicator of soil quality and nutrient supply capacity for C, N and P (Batjes, 2014). In addition, soil C/N, C/P and N/P ratios in natural forests and plantations showed different age patterns with increasing stand age, from which we inferred that Mongolian pine may have different adopt strategies of nutrient utilization and environmental adaptation to sustain plant growth in nutrient‐poor environment (Li et al., 2022). Previous studies have shown that higher soil C/N and C/P ratios imply weaker soil N mineralization and lower P availability (Wang & Yu, 2008), and soil C/P ratio may reflect the fixation efficiency of P to C, and higher C/P ratio has higher C‐fixation efficiency. The observed decline in soil C/P ratios indicates that the C‐fixation efficiency of Mongolian pine was higher in the early growth stage, and then decreased to a large extent during the forest maturation period.

### 4.2 Nutrient resorption and stoichiometric homeostasis among the stand origin

As a plant nutrient conservation mechanism, nutrient resorption is a highly complex process and is used to measure nutrient availability, particularly in the face of nutrient limitation (Killingbeck, 1996; Yang et al., 2018). In this study, litter C, N and P concentrations were lower than those in leaves over all stands, which is consistent with the environmental adaptation of plants (Wang & Zheng, 2020), where leaves transfer nutrients to other growing tissues before senescence and wilting (Cao & Chen, 2017; Schreeg et al., 2014). This process contributes to the maintenance of plant ecological stoichiometric balance and reduces the nutrient loss in forest ecosystems (Deng et al., 2018). This implied that plants in deserts had a higher nutrient utilization efficiency, which is consistent with the view that plants have a higher adaptability under resource‐deficient environment. The data presented here showed that the N and P nutrient resorptions of Mongolian pine in desert regions were lower than global level (62.5% and 64.9%) (Vergutz et al., 2012). This may be due to species differences, with conifers having lower nutrient uptake than herbaceous species. Another possible cause is related to global N deposition, N fertilization increases plant available N and reduces the leaf N nutrient resorption (Yuan & Chen, 2015). Stand origin and stand age appreciable impact on N and P nutrient resorptions, which is due to the huge differences exist in plant photosynthetic capability and nutrient requirements at different growth stages and stand origin (Zhang, Gao, et al., 2018; Zhang, Wang, et al., 2018). In addition, P nutrient resorption in plantations was higher than that in natural forest, indicating that plantations absorbed more nutrients from the litter than natural forest and reduced the absorption of nutrients from the soil. Consistent with the previous studies (Ye et al., 2012), the N and P nutrient resorptions showed an increasing trend in the process of stand maturation, suggesting that soil N and P fertility of the Mongolian pine gradually improved with stand aging. Therefore, mature forests are more capable of replenishing growth‐required N and P via altering the internal N and P cycling in plants (Sun et al., 2019).

Ecological stoichiometry generally presumes that autotrophs have flexible homeostasis (Sterner & Elser, 2002). N and P as the limiting elements, become the main regulators of plant homeostasis. Our study shows that leaf N and P concentrations of natural forests and plantations in the Mu Us Desert were classified as “strictly homeostatic”, suggesting that Mongolian pine has the ability to regulate the balance of elemental demand and nutrient absorption even when limited by the P element, and verifying the mechanism of plant stoichiometric homeostasis (Hessen, 1992; Sterner & Elser, 2002). As expected for the leaf N and P in the Horqin Desert, which were classified as “weakly plastic”, Mongolian pine had N and P homeostasis in natural forest and other plantation. In addition, leaf N/P stoichiometric homeostasis was better for evaluating the homeostasis degree of plants compared with leaf N and P (Blouin et al., 2012). In our study, leaf N/P in plantations were classified as “strictly homeostatic”, which may be because stoichiometric homeostasis was positively correlated with vegetation stability, and leaf N/P ratios were stable among different stand ages. Our results revealed that the degree of stoichiometric homeostasis appeared to differ among stand origins for the same plant (Gu et al., 2017), confirming our second hypothesis, which is due to the complex and variable internal nutrient distribution patterns of plants living in different environments. It is widely accepted that plants with high homeostasis may improve their resilience to drought stress by adopting a more conservative approach to nutrients (Bai et al., 2019; Yu et al., 2010). Overall, although forest ecosystems are highly heterogeneous, they are also highly adaptive. Plants are able to adapt to relatively poor conditions by balancing nutrient cycling and growth with internal nutrient tolerance. This suggests that Mongolian pine is able to prevent adverse environmental conditions such as wind erosion and drought.

### 4.3 Correlation with C–N–P stoichiometry

The above‐ and below‐ground components of the forest ecosystems are tightly coupled, and the feedbacks between them influence ecosystem processes, properties, and functions (Fan et al., 2015). The data presented here indicate that there was a strong coupling relationship between soil nutrients and plant nutrients, which is consistent with precious studies in other forest ecosystems (Qiu et al., 2020; Zhang, Liu, et al., 2019; Zhang, Zhang, et al., 2019). It is easy to understand that C, N, and P concentrations are highest in leaf, then in litter, and follow by soil. The leaf is the photosynthetic organ in plants and requires more N and P elements to meet the needs of a series of metabolic activities during plant growth (Yan et al., 2019). Due to the processes of leaf nutrient resorption and litter decomposition, nutrient concentrations in litter are lower than those in leaves. Litter decomposition is one of the fundamental pathways of nutrient cycling between plants and soils in forest ecosystems (Manzoni et al., 2008). Litter C, N and P stoichiometric characteristics can reflect the efficiency of plant nutrient utilization through root absorb and soil nutrient supply. Soil is not only the basic carrier of plant growth, but also the carrier of litter. It continuously supplies the necessary nutrients (C, N, and P) for plant growth through the root system upwards and absorbs the nutrients released by litter decomposition downwards. Therefore, soil nutrient supply is tightly related to plant growth, development and nutrition metabolism. It is noteworthy that there was no significant correlation between litter and soil P concentration, which can be explained by the large spatial heterogeneity of soil P concentration, and litter P is mainly retained in litter rather than returned to the soil (Robbins et al., 2019). There was a significant negative correlation between soil P concentration and N/P ratio, suggesting that Mongolian pine is mainly limited by P. The low P concentrations in the study regions resulted in an imbalance of N and P, which affected the growth and development of plants, and the material and energy circulation in the forest ecosystem. We also found that leaf N concentration was statistically significantly correlated with leaf N and P concentrations. The reason is that C, N, and P are involved in plant growth and biological processes, such as photosynthetic production, synthesis of protein and nucleic acid synthesis, and these processes require the participation of the C, N, and P nutrient elements.

The ecological stoichiometry of plants is affected by internal and external factors such as developmental stage, soil properties, precipitation, and temperature (Li et al., 2022; Liu & Wang, 2021; Zhang et al., 2017). The RDA results emphasize that the variations in C–N–P stoichiometric characteristics were significantly correlated with environmental factors, and emphasize the contribution degree of soil and meteorological factors in modulating C–N–P stoichiometric characteristics. Consistent with our third finding, the factors influencing C–N–P stoichiometric characteristics varied with stand origin. In addition, we found that soil water content was the most prominent soil factor, water availability is a key driver of N and P cycles, plants decrease the uptake of N and P with declining soil moisture, therefore drought stress will cause a decrease in available N (He & Dijkstra, 2014), leading to low N concentrations in soil and plant. Furthermore, the effect of the soil properties on the C–N–P stoichiometry in the leaf‐litter‐soil system showed that soil water content was distinct positive correlated with soil C and N concentrations. Moisture and soil N are the main limiting factors for plant growth in desert ecosystems (Zhou et al., 2018). The relatively low precipitation and soil water content limit soil microbial activities and N transformation, leading to the reduction of soil C and N concentrations. We also found that soil pH was negatively correlated with soil C concentration. Specifically, higher soil pH generally inhibits soil microbial activity, thereby inhibiting nutrient accumulation in soil (Hu et al., 2018). Litter stoichiometry involves complex interactions between stand age, soil properties, climatic factors, and litter types (Murphy et al., 1998). Stand origin has a significant effect on litter N concentration, owing to the decomposition of litter being greatly influenced by meteorological factors. The difference of climate conditions leads to different rates and degrees of litter decomposition, and the mobility of available nitrogen is strong, which is prone to leaching. Thereby, soil N concentrations were positively correlated with precipitation, and an increase in rainfall will promote litter decomposition and be the return of more effective N to the soil.

## 5 CONCLUSION

Overall, our study showed that the C–N–P stoichiometric characteristics and nutrient resorptions varied significantly among different stand origins, on the contrary, the N/P ratios maintained relatively stable among different stand ages in the leaf‐litter‐soil system. The similar age‐pattern of N, P concentrations and nutrient resorptions were observed in the leaf‐litter‐soil system, which can be described as gradually increasing in the process of stand maturation. Mongolian pine promotes its growth and development promote their growth and development by regulating the balance between elemental requirement and nutrient absorption, and adjusts its nutrient utilization strategies to adapt to the resource‐deficient soil environment in desert regions. We found the significant plant–soil coupling relationships between, indicating that the nutrient elements of C, N, and P are usually in equilibrium within plants. Additionally, Mongolian pine natural forests and plantations in the Hulunbuir Desert and Horqin Desert were limited by N and P, and by P in the Mu Us Desert. Stand origin and stand age impacted the C–N–P stoichiometric characteristics through the variations in climate and soil environment, and the main factors were Pa and SWC. These results could not only improve our understanding of the C–N–P stoichiometry in the leaf‐litter‐soil system with respect to stand origin and stand age but also reveal the balance of C, N, and P nutrient elements in terrestrial ecosystems. However, further studies are needed to investigate the C–N–P stoichiometry of fine roots, which play a major function in nutrient uptake during the whole growth and development of plants.

## AUTHOR CONTRIBUTIONS

**Yue Ren:** Conceptualization (equal); data curation (equal); formal analysis (equal); investigation (equal); methodology (equal); validation (equal); visualization (equal); writing – original draft (equal); writing – review and editing (equal). **Guang‐lei Gao:** Conceptualization (lead); methodology (lead); writing – review and editing (lead). **Guo‐dong Ding:** Conceptualization (lead); writing – original draft (lead). **Ying Zhang:** Methodology (lead). **Pei‐shan Zhao:** Methodology (equal).

## CONFLICT OF INTEREST STATEMENT

The authors declare than they have no conflict of interest.

### OPEN RESEARCH BADGES

This article has earned Open Data, Open Materials and Preregistered Research Design badges. Data, materials and the preregistered design and analysis plan are available at 10.1002/ece3.11172.

## Supporting information


Appendix S1

## Data Availability

We upload the date as supporting material (Appendix S1).
