# Supplementary material for: Patterns and environmental drivers of C, N, and P stoichiometry in the leaf‐litter‐soil system associated with Mongolian pine forests
Source: Ecol Evol. 2024 Mar 20;14(3):e11172. doi: 10.1002/ece3.11172 (PMC10954427; doi:10.1002/ece3.11172)
Supplement: Supplementary file 1 — Appendix S1 [file ECE3-14-e11172-s001.docx]

**TABLE S1** **Correlation coefficients of the C-N-P stoichiometry in the leaf-litter-soil system.**

|  | **Leaf-C** | **Leaf-N** | **Leaf-P** | **Leaf-C/N** | **Leaf-C/P** | **Leaf-N/P** | **Litter-C** | **Litter-N** | **Litter-P** | **Litter-C/N** | **Litter-C/P** | **Litter-N/P** | **Soil-C** | **Soil-N** | **Soil-P** | **Soil-C/N** | **Soil-C/P** | **Soil-N/P** |
| --- | --- | --- | --- | --- | --- | --- | --- | --- | --- | --- | --- | --- | --- | --- | --- | --- | --- | --- |
| **Leaf-C** | - |  |  |  |  |  |  |  |  |  |  |  |  |  |  |  |  |  |
| **Leaf-N** | -0.630^**^ | - |  |  |  |  |  |  |  |  |  |  |  |  |  |  |  |  |
| **Leaf-P** | 0.068 | 0.486^**^ | - |  |  |  |  |  |  |  |  |  |  |  |  |  |  |  |
| **Leaf-C/N** | 0.710^**^ | -0.989^**^ | -0.452^*^ | - |  |  |  |  |  |  |  |  |  |  |  |  |  |  |
| **Leaf-C/P** | 0.395^*^ | -0.669^**^ | -0.993^**^ | 0.667^**^ | - |  |  |  |  |  |  |  |  |  |  |  |  |  |
| **Leaf-N/P** | -0.651^**^ | 0.722^**^ | -0.251 | -0.739^**^ | -0.004 | - |  |  |  |  |  |  |  |  |  |  |  |  |
| **Litter-C** | 0.639^**^ | -0.714^**^ | -0.36 | 0.734^**^ | 0.527^**^ | -0.523^**^ | - |  |  |  |  |  |  |  |  |  |  |  |
| **Litter-N** | -0.193 | 0.502^**^ | 0.631^**^ | -0.486^**^ | -0.609^**^ | 0.066 | -0.690^**^ | - |  |  |  |  |  |  |  |  |  |  |
| **Litter-P** | 0.142 | -0.171 | -0.062 | 0.195 | 0.102 | -0.171 | 0.422^**^ | -0.294 | - |  |  |  |  |  |  |  |  |  |
| **Litter-C/N** | 0.484^**^ | -0.669^**^ | -0.491^**^ | 0.676^**^ | 0.585^**^ | -0.372^*^ | 0.945^**^ | -0.877^**^ | 0.406^*^ | - |  |  |  |  |  |  |  |  |
| **Litter-C/P** | 0.553^**^ | -0.615^**^ | -0.345 | 0.616^**^ | 0.479^**^ | -0.409^*^ | 0.715^**^ | -0.490^**^ | -0.328 | 0.671^**^ | - |  |  |  |  |  |  |  |
| **Litter-N/P** | -0.190 | 0.393^*^ | 0.429^*^ | -0.398^*^ | -0.424^**^ | 0.122 | -0.706^**^ | 0.869^**^ | -0.714^**^ | -0.835^**^ | -0.184 | - |  |  |  |  |  |  |
| **Soil-C** | 0.604^**^ | -0.661^**^ | -0.286 | 0.697^**^ | 0.467^**^ | -0.522^**^ | 0.639^**^ | -0.193 | 0.142 | 0.484^**^ | 0.553^**^ | -0.190 | - |  |  |  |  |  |
| **Soil-N** | 0.323 | -0.172 | 0310 | 0.190 | -0.177 | -0.466^**^ | -0.714^**^ | 0.502^**^ | -0.171 | -0.669^**^ | -0.615^**^ | 0.393^*^ | -0.630^**^ | - |  |  |  |  |
| **Soil-P** | -0.478^**^ | 0.407^*^ | 0.008 | -0.446^**^ | -0.190 | 0.448^*^ | -0.367^*^ | 0.631^**^ | -0.062 | -0.491^**^ | -0.345 | 0.429^*^ | -0.068 | 0.486^**^ | - |  |  |  |
| **Soil-C/N** | 0.381^*^ | -0.567^**^ | -0.434^*^ | 0.567^**^ | 0.536^**^ | -0.293 | 0.734^**^ | -0.486^**^ | 0.195 | 0.676^**^ | 0.616^**^ | -0.389^*^ | 0.710^**^ | -0.989^**^ | -0.452^*^ | - |  |  |
| **Soil-C/P** | 0.523^**^ | -0.709^**^ | -0.388^*^ | 0.722^**^ | 0.549^**^ | -0.488^**^ | 0.527^**^ | -0.609^**^ | 0.102 | 0.585^**^ | 0.479^**^ | -0.424^*^ | 0.395^*^ | -0.669^**^ | -0.933^**^ | 0.667^**^ | - |  |
| **Soil-N/P** | 0.660^**^ | -0.648^**^ | 0.045 | 0.674^**^ | 0.217 | -0.751^**^ | -0.523^**^ | 0.066 | -0.171 | -0.372^*^ | -0.409^*^ | 0.122 | -0.651^**^ | 0.722^**^ | -0.251 | -0.739^**^ | -0.004 | - |

*: significant at *P*<0.05.

**: significant at *P*<0.01.

| (a) | 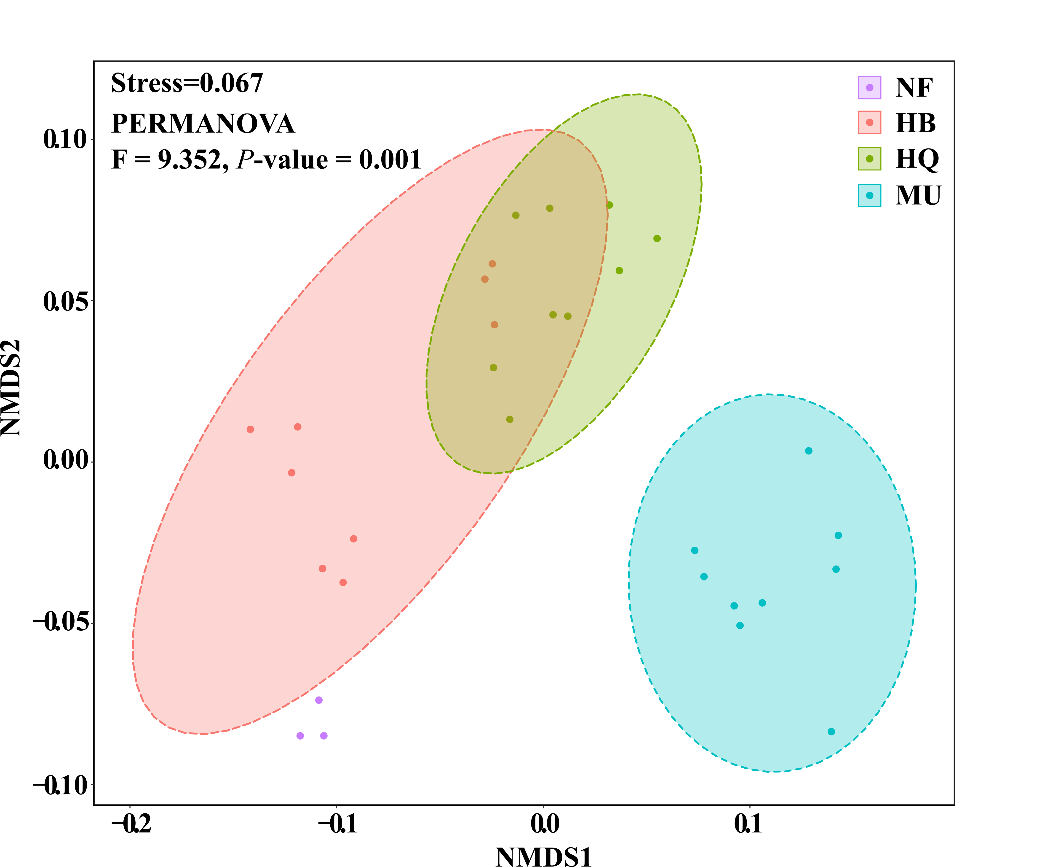 | (b) | 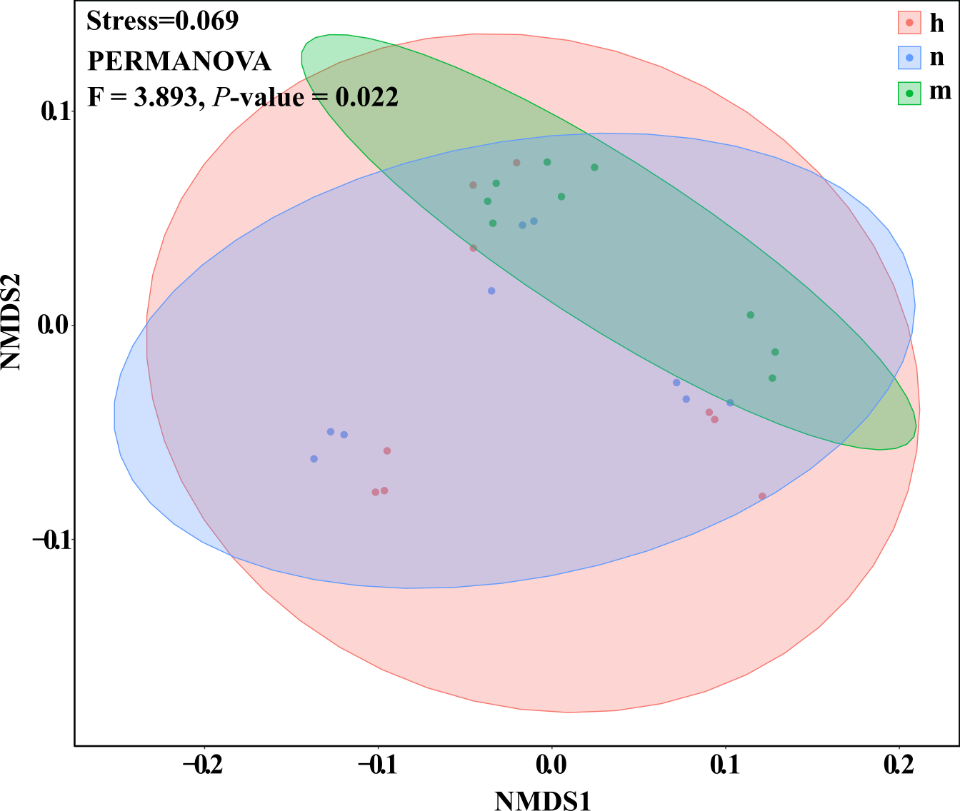 |
| --- | --- | --- | --- |
| **FIGURE S1** **NMDS representations of the differences in the C-N-P stoichiometry.** (a) the effect of stand origin, (b) the effect of stand age. NF: natural forest, HB: the Hulunbuir Sandy Land, HQ: the Horqin Sandy Land, MU: the Mu Us Sandy Land; h: half-mature forest, n: near-mature forest, m: mature forest. The same below. | | | |

**TABLE S2 Constrained ordination of environmental factors.**

| **Factors** | **Order** | **RDA1** | **RDA2** | **r^2^** | ***P* value** |
| --- | --- | --- | --- | --- | --- |
| **Pa** | 1 | 0.953 | -0.302 | 0.801 | 0.0005 |
| **Ta** | 2 | 0.962 | 0.274 | 0.791 | 0.0005 |
| **RHa** | 3 | -0.828 | 0.561 | 0.708 | 0.0005 |
| **SWC** | 4 | -0.738 | 0.675 | 0.696 | 0.0005 |
| **STP** | 5 | -0.281 | -0.960 | 0.518 | 0.0005 |
| **AN** | 6 | -0.988 | 0.155 | 0.516 | 0.0005 |
| **NON** | 7 | -0.999 | -0.031 | 0.447 | 0.0005 |
| **AP** | 8 | -0.984 | 0.180 | 0.415 | 0.0005 |
| **SDa** | 9 | 0.983 | 0.183 | 0.460 | 0.0015 |
| **PHO** | 10 | 0.851 | -0.525 | 0.383 | 0.0020 |
| **pH** | 11 | 0.669 | -0.743 | 0.345 | 0.0045 |
| **INV** | 12 | -0.999 | 0.033 | 0.131 | 0.1439 |
| **URE** | 13 | 0.972 | -0.235 | 0.033 | 0.6327 |

Pa: annual precipitation, Ta: annual temperature, SDa: sunshine duration, RHa: annual relative humidity, SWC: soil water content, STP: soil total porosity, AN: available N, NON: ammonium nitrogen, AP: available P, PHO: phosphatase, INV: invertase, URE: urease. The same below.

**TABLE S3 C, N, and P concentrations and ratios in the leaf-litter-soil system**

| **Component** | **Plot** | **C** | **N** | **P** | **C/N** | **C/P** | **N/P** |
| --- | --- | --- | --- | --- | --- | --- | --- |
| **Leaf** | **NF** | 663.19±2.89 | 34.95±0.45 | 2.26±0.06 | 18.96±0.32 | 293.24±8.38 | 15.47±0.50 |
|  | **HBh** | 671.17±1.40 | 32.87±0.34 | 2.16±0.07 | 20.42±0.24 | 310.65±9.53 | 15.21±0.54 |
|  | **HBn** | 675.70±2.55 | 35.09±1.86 | 2.36±0.08 | 19.31±0.99 | 286.36±8.47 | 14.85±0.35 |
|  | **HBm** | 663.65±0.07 | 36.52±0.49 | 2.44±0.10 | 18.15±0.24 | 271.72±11.09 | 14.67±0.66 |
|  | **HQh** | 663.30±2.35 | 34.60±2.79 | 2.30±0.31 | 19.29±1.53 | 292.68±36.43 | 15.32±2.59 |
|  | **HQn** | 672.91±5.09 | 40.53±3.98 | 2.80±0.24 | 16.76±1.61 | 242.26±18.29 | 14.53±1.33 |
|  | **HQm** | 648.50±0.66 | 46.61±1.19 | 3.01±0.22 | 13.92±0.35 | 216.60±15.29 | 15.57±1.22 |
|  | **MUh** | 622.62±6.00 | 44.65±0.77 | 2.37±0.03 | 13.94±0.23 | 262.11±5.08 | 18.81±0.32 |
|  | **MUn** | 667.53±2.94 | 46.72±0.46 | 2.48±0.07 | 14.29±0.08 | 269.57±8.21 | 18.87±0.61 |
|  | **MUm** | 604.06±3.06 | 49.02±1.93 | 2.48±0.07 | 12.34±0.44 | 244.09±5.86 | 19.81±0.94 |
| **Litter** | **NF** | 456.94±5.58 | 25.71±0.81 | 2.32±0.12 | 17.80±0.75 | 197.42±8.70 | 11.12±0.76 |
|  | **HBh** | 489.12±26.09 | 22.89±0.52 | 0.32±0.11 | 21.41±1.64 | 210.86±4.52 | 9.90±0.72 |
|  | **HBn** | 538.51±3.52 | 23.45±1.13 | 1.96±0.10 | 23.02±1.17 | 275.61±14.07 | 11.97±0.25 |
|  | **HBm** | 456.32±4.97 | 23.96±0.84 | 2.04±0.25 | 19.45±0.90 | 231.08±28.17 | 11.89±1.50 |
|  | **HQh** | 366.19±1.21 | 30.32±2.33 | 1.66±0.10 | 12.15±0.97 | 220.84±11.66 | 18.36±2.28 |
|  | **HQn** | 407.88±7.16 | 35.01±1.05 | 1.94±0.08 | 11.66±0.45 | 210.83±11.66 | 18.12±1.12 |
|  | **HQm** | 345.52±3.62 | 26.94±0.70 | 2.04±0.08 | 9.48±0.08 | 169.75±6.99 | 17.90±0.60 |
|  | **MUh** | 360.08±1.72 | 27.38±0.64 | 1.75±0.06 | 13.37±0.29 | 206.22±7.36 | 15.43±0.62 |
|  | **MUn** | 382.12±1.92 | 29.57±0.17 | 1.95±0.05 | 13.96±0.27 | 195.80±5.24 | 14.03±0.50 |
|  | **MUm** | 343.02±9.50 | 25.71±0.81 | 2.06±0.06 | 11.60±0.39 | 166.76±2.84 | 14.39±0.46 |
| **Soil** | **NF** | 5.38±0.37 | 0.12±0.00 | 0.06±0.01 | 43.80±4.57 | 97.01±14.28 | 2.24±0.38 |
|  | **HBh** | 7.25±0.25 | 0.30±0.02 | 0.13±0.01 | 24.03±2.34 | 57.50±3.60 | 2.40±0.15 |
|  | **HBn** | 11.73±1.18 | 0.53±0.03 | 0.17±0.01 | 22.17±1.00 | 71.10±8.85 | 3.20±0.26 |
|  | **HBm** | 3.12±0.13 | 0.67±0.04 | 0.23±0.01 | 4.64±0.24 | 13.61±0.43 | 2.93±0.09 |
|  | **HQh** | 3.00±0.22 | 0.39±0.00 | 0.11±0.01 | 7.68±0.65 | 27.57±0.45 | 3.61±0.25 |
|  | **HQn** | 3.74±0.06 | 0.43±0.08 | 0.15±0.01 | 9.00±1.92 | 25.75±2.54 | 2.93±0.32 |
|  | **HQm** | 2.71±0.36 | 0.53±0.05 | 0.19±0.01 | 5.25±1.12 | 14.15±2.65 | 2.72±0.16 |
|  | **MUh** | 1.63±0.09 | 0.17±0.01 | 0.82±0.41 | 9.70±0.93 | 3.83±1.09 | 0.38±0.19 |
|  | **MUn** | 2.07±0.56 | 0.22±0.03 | 0.34±0.01 | 9.21±1.47 | 6.07±1.57 | 0.65±0.08 |
|  | **MUm** | 0.72±0.07 | 0.27±0.08 | 0.39±0.03 | 2.86±0.51 | 1.86±0.09 | 0.67±0.14 |

**TABLE S4 Nutrient resorption efficiency of N and P in the leaf**

|  | **NF** | **HBh** | **HBn** | **HBm** | **HQh** | **HQn** | **HQm** | **MUh** | **MUn** | **MUm** |
| --- | --- | --- | --- | --- | --- | --- | --- | --- | --- | --- |
| **N** | 38.82±2.69 | 42.06±1.36 | 44.17±4.75 | 45.39±2.07 | 29.10±0.65 | 35.34±1.48 | 34.94±1.43 | 49.77±2.03 | 51.23±1.18 | 49.73±1.90 |
| **P** | 14.78±2.30 | 10.76±3.19 | 33.75±2.75 | 36.91±1.27 | 39.38±4.30 | 42.10±3.52 | 43.22±5.91 | 38.76±1.48 | 34.40±2.34 | 30.88±0.53 |

**TABLE S5 Soil characteristics of the Mongolian pine across the growth stage between natural forest and plantations**

| **Plot** | **SWC** | **STP** | **pH** | **AN** | **NON** | **AP** | **INV** | **PRE** | **PHO** |
| --- | --- | --- | --- | --- | --- | --- | --- | --- | --- |
| **NF** | 3.97±0.46 | 42.80±0.55 | 6.46±0.22 | 1.99±0.12 | 4.99±0.16 | 2.55±0.15 | 30.94±3.96 | 593.36±16.06 | 0.69±0.45 |
| **HBh** | 9.13±1.22 | 42.72±0.92 | 6.78±0.14 | 46.37±2.52 | 8.78±0.50 | 4.00±0.09 | 70.92±4.90 | 521.83±3.41 | 0.37±0.00 |
| **HBn** | 8.27±0.30 | 45.26±1.70 | 6.94±0.25 | 45.80±1.18 | 7.43±0.72 | 6.30±0.84 | 69.55±0.91 | 329.93±80.62 | 0.38±0.06 |
| **HBm** | 7.20±073 | 42.67±1.70 | 6.81±0.30 | 50.06±2.75 | 8.07±0.43 | 6.21±1.06 | 97.93±2.45 | 457.10±39.7 | 0.56±0.04 |
| **HQh** | 7.51±0.43 | 38.43±0.31 | 6.29±0.31 | 5.81±0.43 | 4.23±0.60 | 2.59±0.24 | 40.29±1.44 | 420.01±22.88 | 0.51±0.07 |
| **HQn** | 7.11±0.45 | 36.97±1.32 | 7.20±0.41 | 4.99±0.13 | 4.09±0.12 | 2.52±0.54 | 51.07±1.50 | 558.40±28.17 | 0.47±0.03 |
| **HQm** | 5.96±0.22 | 36.47±0.35 | 6.42±0.33 | 4.48±0.33 | 4.44±0.59 | 2.17±0.22 | 36.67±2.06 | 567.25±13.81 | 0.65±0.03 |
| **MUh** | 2.95±0.16 | 42.40±0.70 | 7.63±0.25 | 1.02±0.22 | 2.62±0.38 | 2.01±0.18 | 69.61±5.19 | 516.91±34.45 | 0.51±0.04 |
| **MUn** | 3.15±0.30 | 45.80±0.87 | 7.68±0.27 | 1.09±0.22 | 4.84±0.20 | 2.83±0.28 | 58.76±3.78 | 343.56±13.37 | 0.82±0.05 |
| **MUm** | 3.16±0.22 | 45.55±1.62 | 7.63±0.26 | 1.75±0.27 | 5.04±1.24 | 2.49±0.58 | 30.61±2.32 | 531.29±13.15 | 0.79±0.10 |
